# Corneal sensitivity is required for orientation in free-flying migratory bats

**DOI:** 10.1038/s42003-021-02053-w

**Published:** 2021-05-05

**Authors:** Oliver Lindecke, Richard A. Holland, Gunārs Pētersons, Christian C. Voigt

**Affiliations:** 1 Department of Evolutionary Ecology, Leibniz Institute for Zoo and Wildlife Research, Berlin, Germany; 2 AG Verhaltensbiologie, Institute of Biology, Freie Universität Berlin, Berlin, Germany; 3 School of Natural Sciences, Bangor University, Bangor, Gwynedd UK; 4 Faculty of Veterinary Medicine, Latvia University of Life Sciences and Technologies, Jelgava, Latvia

**Keywords:** Animal behaviour, Visual system

## Abstract

The exact anatomical location for an iron particle-based magnetic sense remains enigmatic in vertebrates. For mammals, findings from a cornea anaesthesia experiment in mole rats suggest that it carries the primary sensors for magnetoreception. Yet, this has never been tested in a free-ranging mammal. Here, we investigated whether intact corneal sensation is crucial for navigation in migrating Nathusius’ bats, *Pipistrellus nathusii*, translocated from their migratory corridor. We found that bats treated with corneal anaesthesia in both eyes flew in random directions after translocation and release, contrasting bats with a single eye treated, and the control group, which both oriented in the seasonally appropriate direction. Using a Y-maze test, we confirmed that light detection remained unaffected by topical anaesthesia. Therefore our results suggest the cornea as a possible site of magnetoreception in bats, although other conceivable effects of the anaesthetic are also explored. Furthermore, we demonstrate that the corneal based sense is of bilateral nature but can function in a single eye if necessary.

## Abstract (web summary)

Lindecke and colleagues experimentally investigate the effect of corneal anesthesia on bat migratory orientation. Their results suggest that the cornea is a promising organ for the search of biological magnetic compass sensors, and also indicate that a single functional cornea is sufficient for migratory navigation.

## Introduction

While the capacity for magnetoreception among mammals is evident from a number of behavioural experiments^1–7^, the anatomical location of the involved receptors remains as enigmatic as in any other animal to date^8,9^. Interestingly enough, when tested in darkness, mammals^10–13^, fish^14,15^ and sea turtles^16^ were able to orient by a magnetic polarity compass. The underlying magnetic sense is hypothesized to involve intra-cellular iron oxide, i.e., magnetite nanoparticles (Fe_3_O_4_), which would be sensitive to the horizontal polarity of a magnetic field, enabling these animals to distinguish between magnetic north and south, independent of light. Intra-cellular iron oxide could also be responsible for magnetic signal transmission through control of ion channels depending on the alignment of animals in relation to the magnetic field^8,15,17,18^. Wegner and colleagues postulated that the cornea may be the location of the primary magnetoreceptors in mammals^19^. Specifically, they showed that in mole rats, *Fukomys anselli*, bilateral anaesthesia of the cornea resulted in randomly oriented nest-building, contrary to the usually magnetic polarity-dependent nesting behaviour^10,19^. According to the innervation of the cornea, the ophthalmic branch of the trigeminal nerve would transmit the magnetic signal to the midbrain where magnetic stimuli could be processed^11,13,20–22^. Yet, to date, the hypothesis of a corneal magnetic sense has never been challenged nor expanded from laboratory conditions to freely moving animals by performing a true navigation task in the field, e.g., during seasonal migration.

Non-migratory bats are known to possess a polarity-sensitive magnetic compass, which they use for homing tasks^5,12,23^. Furthermore, results from a classic ‘Kalmijn-Blakemore’ pulse re-magnetization experiment in big brown bats (*Eptesicus fuscus*) are consistent with the hypothesis that magneto-sensory cells located somewhere in a bat’s body carry single-domain magnetite^24^. In contrast, the compass cues and sensory structures the migratory bats use for long-range in-flight navigation still remain undetermined. Only recently, it was demonstrated that bats calibrate their compass system to the solar azimuth at sunset and could take up a seasonally appropriate migratory heading after moderate displacement from their migration corridor^25,26^. To study the role of the cornea in navigation of vertebrates that are adapted to long-range navigation, we performed translocation experiments with 80 adult Nathusius’ bats (*Pipistrellus nathusii*) caught at the Baltic Sea during the late summer migration season. A geographical displacement of the bats was necessary to study their individual orientation behaviour at an unfamiliar site after astronomical twilight and when remote from the high density of conspecifics along the migration corridor, as well as the landmark cues emanating from the shore over short range. Half of the bats received either unilateral or bilateral topical corneal anaesthesia prior to release, while the other half were treated with a saline solution to create a sham control condition. Importantly, we also conducted tests of photoreception in another 76 bats using a Y-maze choice experiment to validate retinal function; specifically, the capacity of bats to still discriminate between light and dark in their environment, despite corneal anaesthesia, was tested.

We hypothesized that migratory bats depend on corneal magnetoreception for navigation. If the cornea plays a role in magnetic orientation, we predicted that translocated bats treated with a topical anaesthetic on both eyes would vanish in random directions after release. However, bats with a single eye treated would be able to navigate because the other eye’s cornea would still be functional, i.e., to transmit sensory stimuli through the ophthalmic branch of the trigeminal nerve, and to enable the released bats to fly in a correct migratory direction similar to bats of the sham treated group.

## Results

### Detection of a light source is unimpaired after topical corneal anaesthesia

In previous Y-maze experiments with one dark and one lit exit, the bats chose the lit exit instead of the dark one^27^. In contrast, blindfolded bats totally deprived of light perception chose the exits randomly^28^. We performed similar tests, yet without blindfolding, to evaluate our bats’ principal ability of light detection after administering topical corneal anaesthesia by oxybuprocaine eye drops. When we tested the unilateral and bilateral treatment groups that received the topical corneal anaesthetic, and the two sham control groups that received eye drops of saline solution bilaterally, our tests did not indicate a differential effect between these applications on the bats’ phototactic behaviour, i.e., animals of both the treatment groups and the sham control groups preferred the lit exit of the Y-maze (sham control 1: *n* = 22, 77% (proportion of bats choosing the lit exit in %), *χ*^*2*^ = 6.55, *W* = 0.55, *P* = 0.011; single eye treated: *n* = 16, 81.3%, *χ*^*2*^ = 6.25, *W* = 0.625, *P* = 0.0124; sham control 2: *n* = 16, 75%, *χ*^*2*^ = 4.0,*W* = 0.5, *P* = 0.046; both eyes treated: *n* = 22, 86%, *χ*^*2*^ = 11.64, *W* = 0.727, *P* < 0.001). Further, exit latency did not differ between bats with bilateral corneal anaesthesia and the respective sham control (both eyes treated, mean ± SD: 11.9 s ± 18.9 SD, median: 4.0 s; sham control 1, mean ± SD: 11.1 s ± 10.7 SD, median: 7.5 s; Mann–Whitney *U*-test: *n* = 44, *U* = 199.5, *P* = 0.321).

### Cornea sensation is crucial for accurate navigation after translocation

**Fig. 1:**
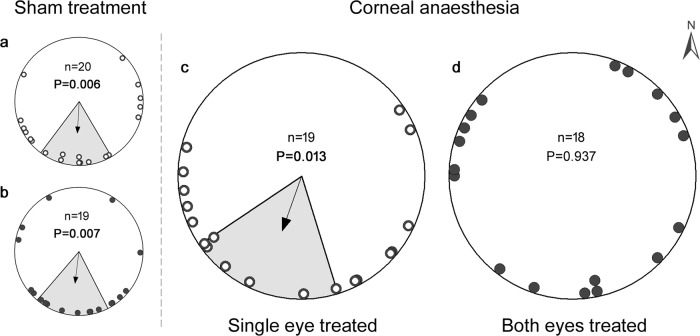
Migratory Nathusius’ bats vanish in random directions if corneal sensation is inhibited in both eyes. **a** and **b** show control bats that received eye drops of saline solution as a sham treatment before release. **c** Experimental bats that randomly received a topical anaesthetic to the left or right eye’s cornea and sham treatment for the other eye, accordingly. **d** Bats with bilateral topical corneal anaesthesia. Empty and filled dots indicate animals that were tracked on the same nights. Arrows depict the group mean vectors in non-randomly oriented groups of bats with the magnetic North (0°) always on top of all plots. Grey sectors encompassing the group mean vectors indicate the 95% confidence intervals for the mean. *P*-values from Rayleigh tests are shown. Total sample size: *n* = 76.

Nathusius’ bats with their eyes untreated were previously shown to spontaneously vanish in a southerly, seasonally appropriate direction after experimental translocation during migration^25,29^. Here, we tested bats caught during their late summer migration at the Latvian Baltic Sea coast. The vanishing bearings of the two sham control groups were also significantly oriented towards the south (Rayleigh’s test, sham control 1, Fig. 1a: mean vector orientation 183° ± 34° (95% confidence intervals), *n* = 20, *r* = 0.495, *Z* = 4.91, *P* = 0.006; sham control 2, Fig. 1b: 187° ± 34°, *n* = 19, *r* = 0.502, *Z* = 4.78, *P* = 0.007) and the circular distributions obtained were best described by unimodal orientation models (Table 1).

**Table 1:** Model-based analysis of bat orientation.

Model	Sham single eye		Sham both eyes		Anaesthesia single eye		Anaesthesia both eyes	
	ΔAICc	*w*	ΔAICc	*w*	ΔAICc	*w*	ΔAICc	*w*
M1 (uniform)	5.81	0.02	5.51	0.02	4.14	0.05	0.00^a^	0.72^a^
M2A (unimodal)	0.00^a^	0.41^a^	0.00^a^	0.35^a^	0.00^a^	0.40^a^	4.67	0.07
M2B (symmetric modified unimodal)	1.13	0.23	0.04	0.35	2.06	0.14	4.67	0.07
M2C (modified unimodal)	3.41	0.07	2.41	0.11	3.72	0.06	6.53	0.03
M3A (homogenous symmetric bimodal)	8.27	0.01	7.81	0.01	7.09	0.01	4.68	0.07
M3B (symmetric bimodal)	4.01	0.05	3.03	0.08	5.35	0.03	7.51	0.02
M4A (homogenous axial bimodal)	5.98	0.02	3.98	0.05	6.65	0.01	7.54	0.02
M4B (axial bimodal)	7.13	0.01	5.70	0.02	8.40	0.01	8.93	0.01
M5A (homogenous bimodal)	2.18	0.14	6.17	0.02	1.53	0.19	10.86	0.00
M5B (bimodal)	4.96	0.03	8.95	0.00	3.03	0.09	13.80	0.00

*w* = AICc model weights.

^a^ Best model to describe the circular data distribution.

There was no difference between the mean orientations and the variances around the mean vector in the two sham control groups (Mardia–Watson–Wheeler test, *W* = 0.189, *P* = 0.91). Bats of the experimental group that received corneal anaesthesia in one eye and sham treatment for the other also vanished in a southerly direction (Rayleigh’s test, single eye treated, Fig. 1c: 199° ± 37°, *n* = 19, *r* = 0.469, *Z* = 4.183, *P* = 0.013). Hence, the group mean vector did not differ from the mean of the respective sham control group (Mardia–Watson–Wheeler test, *W* = 1.011, *P* = 0.603). The variance of individual orientations around the mean also did not differ between the unilateral treatment group and the sham control one from the same migration season (Levene’s test, *F*_1,37_ = 0.224, *P* = 0.639). In contrast to all other groups, bats released with bilateral topical corneal anaesthesia departed in random directions (Rayleigh’s test, both eyes treated, Fig. 1d: 240°, *n* = 18, *r* = 0.061, *Z* = 0.066, *P* = 0.937) and their circular distribution was best described by the uniform orientation model (Table 1). This lack of a preferred direction was distinguishable from the orientation of the control group (*p* < 0.001: the bootstrapped 99.9% confidence interval for the *r*-value from the bilateral sham control group was 0.09 < *r* < 0.86, which does not overlap with the *r*-value of 0.06 in the bilateral anaesthesia group) and also significantly different from the other treatment group (*p* < 0.001: the bootstrapped 99.9% confidence interval for the *r*-value from the unilateral anaesthesia group was 0.14 < *r* < 0.81, which does not overlap with the *r*-value of 0.06 in the bilateral anaesthesia group). The variance of individual orientations between bats that received bilateral anaesthesia and the respective sham control differed significantly (Levene’s test, *F*_1,35_ = 5.824, *P* = 0.021). In addition, the variances around the means of the two groups that received corneal anaesthesia differed (single eye treated *vs*. both eyes treated: Levene’s test, *F*_1,35_ = 5.310, *P* = 0.027).

Experimental and sham control bats vanished promptly from the release site (mean values ± SD, single eye treated: 19.3 min ± 6, median=20 min; sham control 1: 16 ± 6 min, median=14.5 min; both eyes treated: 17.3 min ± 7, median=19.5 min; sham control 2: 16.1 min ± 6, median=16 min). Groups did not differ in the lengths of vanishing times (analysis of variance[ANOVA], *F* = 1.203, d.f.= 3, *P* = 0.135).

## Discussion

To our knowledge, these experiments are the first to elicit a response in the navigation behaviour of a free-ranging mammal migrant without manipulating any sensory cues of the surrounding environment. Further, these data support, for the first time, the hypothesis of an orientation system in bats that relies on corneal sensitivity. Although direct evidence that this is an effect on the magnetic sense in this species is not yet available, it is consistent with previous work from microphthalmic mole rats, which suggests that such a sensory system could be part of a magnetic sense in mammals^19^. Briefly, when bats of our sham treatment groups were released after translocation from their migration corridor, flights were oriented in a seasonally appropriate migratory direction, which is in line with previous data from the same study location^25^. The same was also true when bats were deprived of corneal sensation in only one of their eyes. Yet, with both corneas made temporally insensitive, bats vanished in random directions but at the same speed as other bats. Our Y-maze study shows that under corneal anaesthesia the photoreceptive function of the retina was not neutralized, which meant that the bats could discriminate between light and dark. For take-off, bats would see enough to crawl out of the apparatus and through the preferred lit exit. Thus, upon release, free-ranging bats could have used some visual cues, yet the cornea-anaesthetized bats did not seem to use any visual cues that would enable them to pick their migratory direction. Similar disruption of orientation, independent of retinal impairment, has also been observed in migrating birds and in experiments with homing pigeons, when these encountered magnetic anomalies or fluctuations of the Earth’s magnetic field^30–34^. Also, domestic dogs abandoned their directional preferences for magnetic body alignment during excretion when the rate of change in declination of the Earth’s magnetic field changed^35^. Such disorientation responses were associated not only with an impaired magnetic compass but also with a malfunction of the “map sense” in animals, i.e., when they cannot obtain positional information^17^. This is supported by pigeons that were unable to compensate with other intact compass systems, such as a sun compass, when released in magnetic anomalies^30,32^. Recent evidence supports a “magnetic map sense” in birds based on magnetic iron particles that transmit magnetic field information through the trigeminal system^36–38^. Interestingly, such magnetic particles (magnetite) have also been found in the heads of different bats^39–41^, yet no physical link to any sensorial neuronal network has been established so far. However, magnetic pulsing, which should trigger re-magnetization of any magnetite-based sensor and, thus, provide directionally reversed magnetic compass or map information, led to deflections in adult homing bats that have established a map of their home range^24^. In migratory songbirds, disruption of the magnetic map sense (but not the magnetic compass^42^) can be elicited by bilaterally cutting the ophthalmic branch of the trigeminal nerve^37,38,43^, which is the same branch whose sensation we blocked here through anaesthesia of the corneal nerve endings. Finally, magnetite particles are considered to support a magnetic polarity compass that is independent of light, which was observed in mammals, and also in bats^2,12^.

Another possibility is that if Nathusius’ bats possess a star compass, the observed disorientation in the both-eyes treatment group would suggest side effects of the topical anaesthesia on their vision, e.g., on their visual acuity and, thus, the capacity to discriminate between stars in the sky. Although this possibility cannot be entirely excluded, two lines of evidence suggest it is a less likely explanation for our results. First, the anaesthetic oxybuprocaine is routinely used in mouse models for vision research where a stable ocular pressure and full retinal function are a prerequisite^44–50^. Side effects of the anaesthetic therefore are unlikely, but this alternative requires further testing in bats specifically. Second, although we do not have direct evidence for a magnetic compass in this species, no experiment on the compass system in any bats, or indeed any mammal, has yet provided positive evidence for a role for the stars: either as their primary mechanism of orientation, or as a calibration reference^5,23,26^.

Future studies need to clearly establish the role of the magnetic sense in migratory bat navigation, and focus on the location and, particularly, the cellular mechanisms behind any trigeminal magnetosensor^51^. However, as nocturnal animals, bats have relatively large corneal surfaces and the cornea ranks among the most densely innervated tissues in the mammalian body, which renders it a promising organ for the search of biological “compass needles”^19,21,52–54^.

## Methods

### Animal subjects

All work was conducted under the permits #10/2015, #31/2016, #33/2017-E and #3.6/85/2017-N-E issued by the Latvian Nature Conservation Agency to the Institute of Biology, University of Latvia. Over the course of three field seasons, we captured 156 adult Nathusius’ bats (*P. nathusii*), using a custom-made directional funnel trap (35 × 50 × 15 m; length × width × height) set up adjacent to the shoreline of the Baltic Sea at Pape Bird Ringing Station (PBRS; 56°09' N 21°03' E, Rucava Municipality, Latvia). Capture effort was most intense during the peak of the late summer migration season (between 14 Aug to 1 Sep 2015, 19 Aug to 23 Aug 2016, and 18 Aug to 4 Sep 2017). Bats were aged based on the closure of the epiphyseal gaps. While bats assigned to the retina function test (*n* = 76) were only controlled for seasonally appropriate body mass (≥7.0 g), individuals assigned to the translocation experiment (*n* = 80) were also transitionally ringed and measured for body mass and forearm length. Subsequently, animals were transferred to a keeping facility, where they were kept in groups of up to five individuals in wooden boxes (38 × 19 × 13 cm^3^) in a dark and quiet environment, simulating a natural daytime roost in a tree hollow. Each evening the animals were fed. The duration of animal maintenance ranged from 2 to 5 days to secure, suitable release conditions for experimental nights (relatively high ambient temperature, no rain and low wind conditions). The retinal function experiments were conducted indoors and on the night subsequent to the capture of bats. Animals were housed in small groups and had no access to the natural night sky before release. Captive bats were fed individually with mealworms (larval stages of *Tenebrio molitor*, Coleoptera) during the evening hours and provided ad libitum water. Prior to feeding on experimental evenings, bats also received three small drops of saline solution (NaCl) into the nostrils, as they served as a control group for another study, which, however, did not require any additional experiments for the individuals of this study. That way, we also guaranteed blind study procedures. We do not expect an effect on visual performance and corneal sensation from this nasal treatment.

### Topical anaesthesia of the cornea

Bats were gently held in an upright position and treated with one drop of oxybuprocaine hydrochloride (0.4%, Novesine®, Novartis, Germany) to the central cornea using a pipette. We chose this topical anaesthetic over lidocaine, which is commonly applied in studies of orientation physiology, because lidocaine is known to occasionally cause ophthalmic side effects in birds and mammals, including visual impairment, when penetrating deep into tissues, potentially crossing the blood–brain barrier^51,55–57^. Oxybuprocaine is different to lidocaine as it numbs only the outermost layers of the cornea while leaving the retina unaffected; however, its anaesthetic efficiency decreases after 30 min and thus the sensory impairment is quickly reversible^58,59^. For these reasons, oxybuprocaine is therefore routinely used in human and veterinary ophthalmology^44–50,58–60^. As a control, i.e., for a sham treatment, we used eye drops of sterile saline solution (NaCl 0.9%, B. Braun Melsungen AG, Germany), which is a standard in both human and veterinary ophthalmology, and eye care^59^.

The bilateral treatment group received corneal anaesthesia to both eyes. Bats from the unilateral, i.e., single-eye treatment group, and control bats (sham control groups 1 and 2) received a drop of the sham treatment to the contralateral or both eyes, respectively. After 20 s of exposure, any supernatant was gently removed from the surface of the eye using sterile tissue, and only then the contralateral eye was treated. It is noteworthy that bats neither blinked during this procedure, nor did they show any signs of discomfort, such as emitting of distress calls or spontaneous movements. The choice for the individual cornea treatment was made in a blinded fashion, with students assisting the experimenter. The experimenter received two identical unlabelled pipettes and a note on lateral allocation for the application of eye drops. The left–right ratio of the unilateral treatment was kept at balance over the course of the study period, yet lateral allocations followed a randomized order each night. Behavioural testing started immediately after the eye drops were applied. Bats assigned to the navigation experiment received the treatment only after translocation, just before individual releases.

### Testing retinal function and phototactic behaviour

To make sure topical corneal anaesthesia did not completely abolish the bats' visual capability, we tested 76 bats for phototactic responses in a Y-maze task. Bats are known to choose lit exits over dark ones for emergence from Y-mazes^27,28^. We compared the bilateral anaesthesia treatment (*n* = 22; 14/8 males/females) with a sham control (*n* = 22; 10/12; “sham control 2”) in 2015, and the unilateral treatment (*n* = 16; 5/11) with another sham control (*n* = 16, 5/11, “sham control 1”) in 2017. Tests were performed indoors at PBRS, at room temperature, and were performed between 0300 and 0600 h over the course of two nights in both years. Experimental individuals were kept in wooden boxes until tested. The maze apparatus was made out of plywood, and was inclined towards the exits 10° following recommendations of previous works^28^. The Y-maze had an arm length of 200 mm; cross-sectional dimensions of the runway were 80 × 60 mm^2^ (width × height). All surfaces were coloured dark-brown to minimize light reflections. For the floor, an easy-to-clean PVC coating with a structured surface was used, which was not slippery for crawling bats. The entrance of the Y-maze had a light level of 0.02 lx. Dim light (120 lx) was provided at the exit of one arm using three commercial white torch LEDs indirectly illuminating the space behind the exit, while the exit of the other arm was kept dark (0.01 lx). The area of the bifurcation inside the Y-maze was illuminated indirectly (0.12 lx) via the lit arm. Each bat was transferred manually to the acclimatization compartment of the Y-maze, directly after corneal anaesthesia or sham treatment, respectively. Besides the Y-maze illumination, the testing room was kept dark. After 20 s for acclimatization, a bat had to crawl a 100 mm runway to reach the bifurcation. We timed the emergence latency. Bats of both groups were tested in alternate order, with the lit arm of the maze changed after the first half of bats had been tested. Clean sheets soaked with ethanol (70%) were used to clean the runways between trials. Individuals were tested only once and released in the nearby coastal forest after 1 h to ensure that anaesthesia had ceased before bats were free again. When dawn was approaching, bats were kept for the next day, fed and watered in the evening and released immediately after that at the site of capture. Emergence latency was compared using the Mann–Whitney *U*-test since data were not normally distributed (*P* < 0.05). Directional choices for exits of each group were analysed for a preference using a test of goodness of fit (chi-squared test; R version 3.2.1, package *shiny*).

### Testing navigational performance after translocation and corneal anaesthesia

We used 80 adult *P. nathusii* (36 males, 44 post-lactating females) for the release experiment. On the day of the translocation, bats were fed and watered from 1800 to 2000 h. Subsequently, they were equipped with VHF radio transmitters (operating frequency wavelengths: 150.00–152.00 MHz; LB-2XT, Holohil Systems Ltd., Ottawa, Canada, 0.31 g; V1 and V3, Telemetrie-Service Dessau, Dessau-Roßlau, Germany, 0.35 g; Pip Ag337 and PicoPip Ag379, BioTrack Ltd., Wareham, UK, 0.35 and 0.43 g). One radio transmitter was glued onto the fur of the lower dorsum of each bat using skin glue (Manfred Sauer GmbH Hautkleber, Lobbach, Germany). Transmitters were selected so that the mass of the tag was lower than 5% of the individual body mass. Until translocation to the release site, bats were kept individually in large cloth bags to allow acclimatization to the tag. Translocation and releases were performed between 2300h and 0400 h of a given night and over the course of 26 nights. The release site was a flat field about 11 km east of the capture site and outside the coastal migration corridor where bats were caught. The location offered a clear line of sight of the horizon for 360°. To increase the motivation to continue migratory transit flights, bats were offered water and mealworms again prior to release but before any cornea treatments. The person who tracked the animals was blind to the treatment conditions. To achieve this, the assisting personnel randomly chose the substances to be applied, i.e., chose the test group, and consequently provided the experimenter with one pipette per eye for applications. Thereby, we ensured unbiased measurements of vanishing bearings. We aimed to release an even number of bats per group and per night. Only the assisting personnel tracked the sequence of experimental and control bat releases on a given night and could balance the number and succession of releases of both groups of treatments. Before treatment and release from the roof of the car, we surveyed the vicinity of the site for the presence of any other bats using a bat detector (Echometer EM3 + , Wildlife Acoustics, Inc., Maynard, MA, USA). If any bat would have been recorded, the experiment would have been paused to avoid confounding via eavesdropping. After the cornea treatment and prior to releasing, the surrounding was surveyed for bats again for 1 min. In the absence of bat activity, test bats were offered to take-off at their own speed while the release direction was chosen randomly. Bats were then tracked at about 4 m above the ground using a handheld three element Yagi antenna attached to an Australis 26k receiver (Titley Scientific). When the signal of the radio transmitter had vanished, the bearing of the fading signal and the time elapsed since the release was noted. After 2 min, we confirmed the absence of bats by monitoring the area for the individual radio signal again. This was also repeated for all individuals of the given night after the last bat had vanished. The next night, a complete scan for all frequencies was repeated before any new bat was released. For statistical comparisons we did not include data from bats that took >30 min for vanishing (*n* = 4) because the full efficiency of the corneal anaesthesia lasts for half an hour^44,47,49,58,59^. Also, in a previous study, *P. nathusii* vanished from the tracking range in less than 20 min from the same release site^25^, indicating that significantly longer vanishing times most likely represent outliers.

The mean bearings and vector lengths of each group were calculated using the Oriana 4.02 circular statistics software package (Kovach Computing Services). Groups were tested for departure from a uniform circular distribution using the Rayleigh’s test^61^. In order to further evaluate the distribution of bearings of our experimental groups, in particular the pattern of the non-unimodally oriented group with bilaterally topical anaesthesia, we applied a likelihood-based modelling approach (package CircMLE, R version 3.5.2) that has recently been introduced to compare circular data with multiple potential models of orientation behaviour^62^. Beyond the uniform distribution representing random scatter of bearings (M1), these models comprise three unimodal variants (ordinary, M2A; symmetric modified, M2B; modified unimodal, M2C) and six bimodal variants of distributions (homogenous symmetric bimodal, M3A; symmetric bimodal, M3B; homogenous axial bimodal, M4A; axial bimodal, M4B; homogenous bimodal, M5A; and bimodal, M5B)^63^. For each experimental group, resulting models were then compared by means of the corrected Akaike information criterion (AICc) and the corresponding model weights^64^. Tests for significant differences between group orientations were performed using the Mardia–Watson–Wheeler test.

For a more sophisticated comparison of the directedness between the bilateral treatment group and the other two significantly unimodally oriented groups (the unilateral treatment group and sham control 2; Fig. 1b, c), we followed a recently introduced bootstrap technique^65^. For this, the mean resultant vectors (*r*-values) of different experimental groups are used to observe whether the *r*-value of a non-significantly oriented group falls within some confidence intervals of another *r*-value that derives from a significantly oriented group. To do so, a random subsample of *n* orientation angles is drawn with replacement from a significantly oriented sample of *n* orientation angles present in the significantly oriented treatment groups (*n* = 19 for both the unilateral treatment group and the sham control 2). Then the corresponding *r*-value is calculated based on these *n* = 19 orientation angles. With a new randomization each time, this procedure is repeated 100,000 times. The resulting 100,000 *r*-values are ranked lowest to highest. The *r*-values at the ranks 2500 and 97,500, 500 and 99,500, and 50 and 99,950 define the 95%, the 99%, and 99.9% confidence limits for the observed *r*-value of the significantly oriented group, respectively. If the *r*-value observed in the actually non-significantly oriented group lies outside these confidence intervals, the significantly oriented group is significantly more directed than the non-significantly oriented group with a significance of *p* < 0.05, *p* < 0.01, or *p* < 0.001, respectively.

Navigational accuracy between groups was assessed by testing for homogeneity of variances across groups, i.e., the scatter of the bearings. For this, the original bearings were transformed to absolute residuals from the group-specific orientation mean. With these we computed a Levene’s test^66^, which does not assume underlying normality of the data (R package *car* version 2, R). Departure flight times were compared using an ANOVA Kolmogorov–Smirnov test for normality passed, *P* = 0.083).

### Reporting summary

Further information on research design is available in the Nature Research Reporting Summary linked to this article.

## Supplementary information

Reporting Summary

## Data Availability

The data that support the findings of this study are available from the corresponding author upon reasonable request.
