# Supplementary material for: Corneal sensitivity is required for orientation in free-flying migratory bats
Source: Commun Biol. 2021 May 5;4:522. doi: 10.1038/s42003-021-02053-w (PMC8100159; doi:10.1038/s42003-021-02053-w)
Supplement: Supplementary file 1 — Reporting Summary [file 42003_2021_2053_MOESM1_ESM.pdf]

nature research

Corresponding author(s): Oliver Lindecke
Last updated by author(s): 12.01.2020

Reporting Summary

Nature Research wishes to improve the reproducibility of the work that we publish. This form provides structure for consistency and transparency in reporting. For further information on Nature Research policies, see Authors & Referees and the Editorial Policy Checklist .

Statistics

For all statistical analyses, confirm that the following items are present in the figure legend, table legend, main text, or Methods section.

n/a	Confirmed	
☐	☒	The exact sample size (*n*) for each experimental group/condition, given as a discrete number and unit of measurement
☐	☒	A statement on whether measurements were taken from distinct samples or whether the same sample was measured repeatedly
☐	☒	The statistical test(s) used AND whether they are one- or two-sided<br>*Only common tests should be described solely by name; describe more complex techniques in the Methods section.*
☒	☐	A description of all covariates tested
☒	☐	A description of any assumptions or corrections, such as tests of normality and adjustment for multiple comparisons
☐	☒	A full description of the statistical parameters including central tendency (e.g. means) or other basic estimates (e.g. regression coefficient) AND variation (e.g. standard deviation) or associated estimates of uncertainty (e.g. confidence intervals)
☐	☒	For null hypothesis testing, the test statistic (e.g. *F*, *t*, *r*) with confidence intervals, effect sizes, degrees of freedom and *P* value noted<br>*Give P values as exact values whenever suitable.*
☒	☐	For Bayesian analysis, information on the choice of priors and Markov chain Monte Carlo settings
☒	☐	For hierarchical and complex designs, identification of the appropriate level for tests and full reporting of outcomes
☒	☐	Estimates of effect sizes (e.g. Cohen's *d*, Pearson's *r*), indicating how they were calculated

Our web collection on statistics for biologists contains articles on many of the points above.

Software and code

Policy information about availability of computer code

Data collection	No software was used.
Data analysis	Oriana 4.02 circular statistics software package (Kovach Computing Services) for descriptive statistics and Rayleight tests and Mardia-Watson-Wheeler tests. R version 3.3.2, package car version 2 for Levene tests, package shiny for Chi-squared test, R 3.5.2 package CircMLE for likelihood-based modelling

For manuscripts utilizing custom algorithms or software that are central to the research but not yet described in published literature, software must be made available to editors/reviewers. We strongly encourage code deposition in a community repository (e.g. GitHub). See the Nature Research guidelines for submitting code & software for further information.

Data

Policy information about availability of data

All manuscripts must include a data availability statement. This statement should provide the following information, where applicable:
- Accession codes, unique identifiers, or web links for publicly available datasets
- A list of figures that have associated raw data
- A description of any restrictions on data availability

The data that support the findings of this study are available from the corresponding author upon reasonable request.

Field-specific reporting

Please select the one below that is the best fit for your research. If you are not sure, read the appropriate sections before making your selection.

Ecological, evolutionary & environmental sciences study design

All studies must disclose on these points even when the disclosure is negative.

Study description	To study the role of the cornea in navigation of vertebrates, and mammals that adapted to long-range navigation in particular, we performed a series of translocation experiments with adult European migratory pipistrelles at the Baltic sea during their migration to the wintering and mating grounds. Half of the bats received either unilateral or bilateral topical cornea anaesthesia prior to release. Importantly, we also conducted tests of retinal function in bats using a Y-maze choice experiment to validate retinal function, specifically the light-dark discrimination ability of bats despite corneal anaesthesia.
Research sample	The bats selected for our experiments represent Nathusius' bats, Pipistrellus nathusii, from the north-eastern populations that migrate across Europe when flying towards their wintering grounds. We chose adult individuals (> 1 year old) of both sexes to guarantee that the test bats are experienced (long-distance) migrants that readily make use of a putative magnetic orientation system.
Sampling strategy	Data acquired during the retinal function test, i.e., the exit choice and time to exit were gathered by visual observation and a hand-held stop watch. For the navigational tests bats were tracked at about 4 m above ground using a handheld three element Yagi antenna. When the signal of the radio transmitter vanished, the bearing of the fading signal and the time elapsed since the release were noted. We conducted a similar experiment on navigation after translocation one year before our study (Lindecke et al. 2015 Biol. Lett.) and based on this experience we aimed to test around 20 bats per experimental group. We note that as analysis of bearings is with circular statistics, formal power analysis is not possible.
Data collection	Any data was collected by author, Oliver Lindecke, who was also the experimenter. After capture, bats were aged based on the closure of the epiphyseal gaps. Bats assigned to the retina function test were only controlled for seasonally appropriate body mass (≥7.0 g), yet individuals assigned to the navigation experiment were measured for body mass and forearm length to ensure that radio tags would not exceed 5% of the individual body masses. Animals were fed and watered and their body mass controlled on each evening in captivity. The collection of data during these checks were also made possible with the help of field assistents. Behavioural testing started immediately after the eye drops were applied. For the retinal function tests, the chosen arm of the Y-maze and exit latency were recorded. Bats assigned to the navigation experiment received the treatment only after translocation, just before individual releases. Vanishing bearings were measured using a handheld compass. Two minutes after the last positive signal detection, the absence of bats was confirmed by monitoring the area for the individual radio signal again. This was repeated also for all individuals of the given night after the last bat has vanished. The next night, a complete scan for all frequencies was repeated before any new bat was released to confirm the previous bat vanishings again.
Timing and spatial scale	Data was collected over the course of three field seasons. Capture efforts were most intense during the peak of the late summer bat migration season between 14 Aug to 1 Sep 2015, 19 Aug to 23 Aug 2016, and 18 Aug to 4 Sep 2017). Once the capture attempts were successful, the experimental testing was scheduled so that release conditions would be favourable according to the wind and weather forecasts. The duration of animal maintenance ranged from 2 to 5 days to secure suitable release conditions. Frequency and periodicity of experiments was therefore weather-dependent.
Data exclusions	We did not consider bats that took >30 min for vanishing in the navigation test (n=4 bats) because full efficiency of the corneal anaesthesia lasts for half an hour only. As this was a known factor with literature evidence, the exclusion criterion was pre-established.
Reproducibility	The techniques used for this work are a combination of methods have been repeatedly applied in other earlier studies. The retinal function test has been successfully repeated for a group of control animals for another study at the same location, with the same species and using the same equipment. The navigational performance test, i.e., radiotracking of translocated bats has been successfully repeated, i.e. with the same result regarding the mean vanishing direction, in a control group for another experiment running in parallel, while this control group even received the exact same treatment (NaCl eyedrops onto the cornea).
Randomization	Assistants were advised to randomize the succession of the different treatments for every night of the study. Further, sexes ratios of bats were kept balanced between treatment groups.
Blinding	Release directions of the bats were chosen randomly and the experimenter who radio-tracked the bats was blind to the treatment. The assistant who decided over the invidual experimental treatment of bats was kept blind to the tracking results. The experimenter who tracked the animals analysed the data after the assistants allocated the individual bats to treatment groups.<br>For both, the retinal function test and the navigation performance study the assisting personnel randomly chose the substances to be applied, i.e., chose the test group, and consequently provided the experimenter with one pipette per eye for applications.

Did the study involve field work? ☒ Yes ☐ No

Field work, collection and transport

Field conditions	Release experiments were conducted only under suitable environmental conditions, i.e., relative high ambient temperature >4° C, no rain and zero to low wind conditions. Translocation and releases were performed between 2300 and 0400 hours of a given night.<br>Before treatment and release of bats, we ensured that no other bats were active in the vicinity of the site by means of scanning for ultrasound calls using a bat detector. If any other free-flying bat would have been recorded, the experiment would have been paused to avoid attraction of the test bats to near conspecifics.

Location	Bats were captured at Pape Bird Ringing Station (56.16° N 21.01° E, 0 m a.s.l., Rucava Municipality, Latvia). The release site for bats that were radio tracked was 11 km east of the capture location at 56.17° N 21.19° E, 21 m a.s.l. The site was a flat field offering a clear line of sight of the horizon for 360°.
Access and import/export	All work with bats at Pape Bird Ringing Station was conducted under the permits #10/2015, #31/2016, #33/2017-E and #3.6/85/2017-N-E issued by the Latvian Nature Conservation Agency to the Institute of Biology, University of Latvia. No biological samples were either imported nor exported.
Disturbance	Bats were gently held in an upright position and treated with one drop of oxybuprocaine hydrochloride (0.4%, Novesine®, Novartis, Germany) to the central cornea using a pipette. This topical anaesthesia temporally impairs the sensation in the corneal tissues. However, the anaestethic effect gets less effective after 30 mins and disappears completely without the need of additional capture of animals.

Reporting for specific materials, systems and methods

We require information from authors about some types of materials, experimental systems and methods used in many studies. Here, indicate whether each material, system or method listed is relevant to your study. If you are not sure if a list item applies to your research, read the appropriate section before selecting a response.

Materials & experimental systems

n/a	Involved in the study
☒	☐ Antibodies
☒	☐ Eukaryotic cell lines
☒	☐ Palaeontology
☐	☒ Animals and other organisms
☒	☐ Human research participants
☒	☐ Clinical data

Methods

n/a	Involved in the study
☒	☐ ChIP-seq
☒	☐ Flow cytometry
☒	☐ MRI-based neuroimaging

Animals and other organisms

Policy information about studies involving animals; ARRIVE guidelines recommended for reporting animal research

Laboratory animals	The study did not involve laboratory animals.
Wild animals	Adult Nathusius' bats (Pipistrellus nathusii) of both sexes were captured during late summer migration using a funnel trap aligned to the Baltic Sea shore (dimensions at the entrance of the funnel 50 x 15 m). After capture bats were put in cloth bags and wooden boxes. Bats were transferred to a dark keeping room where other wooden boxes served as cages for subsequent day-roosting. Transportation to the release site was by car with bats kept in individual cloth bags in wooden boxes. The bats were released directly back into the wild when they were set free from the roof of the car for radio tracking of there migratory flights. Bats that were used to investigate the retinal function were tested at Pape Bird Ringing Station and released back into the wild at the location of capture, i.e., behind the funnel trap.
Field-collected samples	The study did not involve samples collected from the field.
Ethics oversight	Latvian Nature Conservation Agency, Institute of Biology (University of Latvia), Food and Veterinary Service Latvia, Leibniz Institute for Zoo and Wildlife Research

Note that full information on the approval of the study protocol must also be provided in the manuscript.
